# Angustoline Inhibited Esophageal Tumors Through Regulating LKB1/AMPK/ELAVL1/LPACT2 Pathway and Phospholipid Remodeling

**DOI:** 10.3389/fonc.2020.01094

**Published:** 2020-07-07

**Authors:** Huiying Li, Cheng Zhang, Min Zhang, Qianqian Yao, Huaigu Yang, Linlin Fan, Nan Zheng

**Affiliations:** ^1^Key Laboratory of Quality and Safety Control for Milk and Dairy Products of Ministry of Agriculture and Rural Affairs, Institute of Animal Science, Chinese Academy of Agricultural Sciences, Beijing, China; ^2^Department of Cardiothoracic Surgery, First Affiliated Hospital of Chongqing Medical University, Chongqing, China

**Keywords:** esophageal cancer, LKB1, AMPK, ELAVL1, LPCAT2, lipidomics

## Abstract

Esophageal cancer is a type of gastrointestinal carcinoma and is among the 10 most common causes of cancer death worldwide. However, the specific mechanism and the biomarkers in the proliferation and metastasis of esophageal tumors are still unclear. Therefore, the development of several natural products which could inhibit esophageal tumors deserve attention. In the present study, different sources of cancer cells were used to select the sensitive cell line (esophageal cancer cell KYSE450) and the proper dose of angustoline, which were utilized in the following cell viability, migration and invasion assays. Then the lipidomic detection of clinical samples (tissue and blood plasma) from esophageal cancer patients was performed, to screen out the specific phospholipid metabolites [PC (16:0/18:1) and LPC (16:0)]. Considering lysophosphatidylcholine acyltransferase 2 (LPCAT2) was tightly relative with phospholipids conversion, serine/threonine-protein kinase 11 (LKB1), 5′-monophosphate (AMP)-activated protein kinase (AMPK) and embryonic lethal, and abnormal vision, drosophila-like 1 (ELAVL1) were investigated, to evaluate their expression levels in esophageal tumor tissue and KYSE450 cells. Additionally, KYSE450 tumor bearing mouse model was constructed, the role of angustoline in inhibiting esophageal tumors through regulating LKB1/AMPK/ELAVL1/LPCAT2 pathway was validated, and found that the conversion from LPC (16:0) to PC (16:0/18:1) was blocked by angustoline in some degree. The above results for the first time proved that angustoline suppressed esophageal tumors through activating LKB1/AMPK and inhibiting ELAVL1/LPCAT2, which consequently blocked phospholipid remodeling from LPC (16:0) to PC (16:0/18:1).

## Introduction

Esophageal cancer is a type of gastrointestinal cancer and is among the 10 most common causes of cancer death worldwide. Statistical data shows that over 3,000 thousands of patients die from esophageal cancer every year (1). There are two main histological types, squamous cell carcinoma (SCC), which is the predominant histological type worldwide, and adenocarcinoma (ADC). Adenocarcinoma is mainly a disease of developed countries, and the epidemiology of esophageal cancer differs markedly from those of other epithelial cancers. There is a huge variation in its incidence worldwide, with > 100-fold differences observed between high-incidence areas, such as China and Iran, and low-incidence areas, such as western Africa. The average age of patients with esophageal cancer is > 45 years; the proportion of males is higher than that of females; and the typical symptoms of this cancer include progressive dysphagia and severe pain (1).

Research has shown that the etiology of esophageal cancer is associated with age, sex, occupation, region, living environment, dietary habits, and hereditary susceptibility, which suggests that esophageal cancer is a multifactorial disease.

Although the surgical treatments of esophageal cancer are well-established, the specific mechanism of its pathogenesis and especially the biomarkers of the its development, are still unclear. In this study, we utilized lipidomics methods to screen out the specific lipid metabolites in the tumor tissues and blood plasma samples of patients with esophageal cancer. Additionally, the effects of angustoline on viability, migration and invasion of esophageal cancer cells (KYSE450) were evaluated, as well as the regulation of angustoline in LKB1/AMPK/ELAVL1/LPCAT2 pathway in KYSE450 tumor bearing mice was investigated.

## Materials and Methods

### Chemicals and Reagents

The human colorectal cancer cell line (HT29), human esophageal squamous cell lines (KYSE150, KYSE450, ECA109, TE13), human hepatocellular carcinoma cell line (HepG2), human breast cancer line (MDA231), human lung cancer line (A549), and normal esophageal epithelial cell line (HET1A), were purchased from the Chinese Academy of Science (Shanghai, China). Dulbecco's modified eagle medium (DMEM), Roswell Park Memorial Institute (RPMI)-1640 medium, fetal bovine serum (FBS), and penicillin-streptomycin solution was purchased from Gibco (New York, NY, USA). Angustoline was purchased from Sigma (San Francisco, CA, USA), with the purity of 95%. The AMPK activator (AICAR) was purchased from Abcam (Shanghai, China), LKB1 small interfering RNA (siRNA), AMPK siRNA, and LPCAT2 siRNA fragments were synthesized by Sangon (Shanghai, China). Antibodies directed against LKB1, AMPK, ELAVL1, LPCAT2, and β-actin, and the corresponding secondary antibodies, were purchased from Santa Cruz Biotechnology (San Francisco, CA, USA). Other reagents for the western blotting assay were purchased from Solarbio (Beijing, China). The enhanced chemiluminescence (ECL) reagent was purchased from Thermo Scientific (Waltham, MA, USA).

### Cell Culture

HT29 cells, KYSE150, KYSE450, ECA109, and TE13 cells were grown in RPMI-1640 medium containing 10% FBS, HepG2 cells, MDA231 cells, and A549 cells were grown in DMEM medium containing 10% FBS. The growth medium was replaced with fresh medium every day. The cells were passaged every 2 days. All cells were cultured with 1% penicillin-streptomycin in a humidified incubator (Thermo Scientific) at 37°C under 5% CO_2_.

### Detections of Cell Viability, Migration, and Invasion

HT29 cells, KYSE150 cells, KYSE450 cells, ECA109 cells, TE13 cells, HepG2 cells, MDA231 cells, A549 cells, and HET1A cells (1 × 10^4^ cells in 100 μL growth medium per well) were plated in a 96-well plate and incubated for 24 h, respectively. When the medium was replaced with 100 μL fresh medium containing increasing concentrations of angustoline (0, 10 μg/L, 100 μg/L, 1 mg/L, 10 mg/L, 100 mg/L and 1 g/L), and cultured for another 48 h. The CCK-8 kit was then utilized according to the manufacturer. The optical densities at 490 nm were measured using a Microplate Reader (Thermo Scientific). The cell viability = (A test–A blank)/(A control–A blank) × 100%. Through comparing with the cell viabilities under the same dosage among five sources of cells (HT29, KYSE450, HepG2, MDA231, and A549), the sensitive cell line (KYSE450) was selected. Furthermore, through comparing with the viabilities of normal esophageal epithelial cells (HET1A) and four types of esophageal tumor cells (KYSE150, KYSE450, ECA109, TE13), the sensitive cell line (KYSE450) to angustoline was further determined. Then the dosages with the cell viability > 85%, as well as those significantly different from control cells (*p* < 0.05), were selected as the final concentration of angustoline used in the following experiments.

The effect of angustoline on the migratory capacity of the sensitive cell line was detected by transwell. The upper chambers were seeded with 5 × 10^3^ cells in 150 μL serum-free medium and 600 μL of medium containing 10% FBS was added to the lower chambers. Samples of angustoline (final concentrations: 100 μg/L and 1 mg/L) were added to the upper chamber and cells were cultured for 24 h. The top surface of the filter was scrubbed gently with cotton swabs, and the migrated cells on the undersurface were fixed with 15% ice methanol for 20 min, then stained with 0.1% crystal violet for 15 min prior to washing with ice PBS buffer (3 min × 3). The stained cells were then photographed and counted, the mean number of stained cells was calculated in three random fields on each undersurface, and the number of migrated cells in the control and treatment groups were compared and analyzed.

The effect of angustoline on the invasion of the sensitive cell line was detected by scratch analysis. Cells were plated in a 6-well plate and incubated for 24 h to achieve a cell density > 85%. A single lesion ~3.0 mm wide was scratched across the cell monolayer by mechanical scraping. The cells were then incubated with angustoline (final concentrations 1 mg/L), and the width of the scratch wound was photographed and scanned 24 h later. The scratch width at the timepoint of 0 h was chosen as the primary scratch width (control 0 h), and the scratch width in the treatment groups represented the inhibitory activity of angustoline on cell invasion. The recovery rate (%) = the scratch width of the denuded area in the treatment groups / the scratch width of the denuded area in the control group (0 h) × 100%.

### Clinical Samples Collection

From May 2018 to January 2019, 30 patients with esophageal cancer were enrolled and treated surgically in this study. Patients' characteristics are reported in Table 1. All patients gave their informed written consent to use biological specimens for investigational procedures, according to the Ethics Committee approval of the First Affiliated Hospital of Chongqing Medical University. The site of anastomosis was selected according to the location of the tumor: cervical manual anastomosis for tumors located in the upper one-third of the esophagus and stapled intrathoracic anastomosis for tumors located in the lower two-thirds of the esophagus. The gastric tube was formed from the distal aspect of the lesser curvature of the stomach using linear staplers by resecting the lesser curvature of the stomach. The formation of the gastric conduit (about 3 cm in diameter) ensured the preservation of the gastroepiploic vessels of the greater curvature of the stomach. Irrespective of the site of anastomosis, all the gastric tubes were placed in the posterior mediastinum. The following tissue samples were taken from the patients with esophageal cancer: the esophageal tumor tissue, as well as the adjacent paracancerous tissue, between which the distance was at least 5 cm, was regarded as the normal tissue. Blood samples of the patients were taken upon admission on the morning of the surgical operation day. As the normal control group, 30 healthy people were selected and enrolled in the study. Their blood samples were collected upon admission in the morning.

**Table 1:** Patients' clinicopathological characteristics.

	**ESCC**	**Normal**
Cases	30	30
**Sex, n (%)**
M/F	26/4 (86.7/13.3%)	22/8 (73.3/26.7%)
**Age**
Mean (years)	64.4	55.93
Median (years)	64	59
**pTNM stage, n (%)**
I	2 (6.6%)	
II	14 (46.7%)	
III	11 (36.7%)	
IV	3 (10%)	

### Lipidomics Detection and Data Analysis

Cell samples (1 × 10^7^ per dish) were treated with 200 μL of isopropanol, vortexed (10 s), and sonicated (5 min). Tissue samples (50 mg) including esophagus tissue, tumor tissue from xenograft mouse and patients were treated with 500 μL of isopropanol, vortexed (20 s), and sonicated (15 min). The above mixture samples were frozen at −20°C for 1 h and then centrifuged (12,000 g, 10 min). The upper layer was collected and transferred to a sample vial to be injected and analyzed with ultrahigh-performance LC-(UPLC)-QTOF-MS, as described in Supplementary Material.

The high-accuracy MS data were recorded with the MassLynx 4.1 software (Waters). The raw data were imported into the commercial software Progenesis QI (version 2.4, hereinafter referred to as “QI”) for processing, which included peak annotation and normalization. Lipids were identified with a database search with the Progenesis QI software (Waters). The differences in all the lipid species between groups were analyzed, and the concentration of each lipid species was compared with the non-parametric Wilcoxon rank-sum test (R package, Robert Gentleman and Ross Ihaka, Auckland, New Zealand) to determine the difference in each metabolite between two groups. A multivariate statistical analysis was performed with OPLS-DA and the VIP of each lipid species was calculated (R package). The relevant false discovery rate (FDR) based on the *p*-value was calculated, and *p* < 0.05 and VIP > 1 were considered to indicate a significant difference. The differential lipid species were graphed with a box plot in GraphPad Prism 7.0.

### Lipid Quantities Determined With LC-MS/MS

The lipid contents of the special lipid metabolites were determined, including PC (16:0/18:1) and LPC (16:0). The standard compounds were obtained from Avanti® Polar Lipids (St. Louis, MO, USA), which is a Sigma company. The 1 μL samples were injected into the LC-MS/MS apparatus (Xevo® TQS, Waters) using an amide column (Acquity UPLC BEH Amide Column, 130 Å, 1.7 μm, 2.1 mm × 100 mm). Mobile phase A was acetonitrile/isopropanol (1:1) containing 10 mM ammonium formate and 0.1% formic acid; mobile phase B was acetonitrile/H_2_O (1:1) containing 10 mM ammonium formate and 0.1% formic acid. The gradient conditions were: 0–1 min, 0.1–70% B; 1–3.5 min, 70–90% B; 3.5–3.6 min, 90–0.1% B; 3.6–5 min, 0.1% B. The flow rate was 0.3 mL/min. The column temperature was 45°C and the sample room temperature was 10°C. Data were acquired in both positive ion modes. The mass spectrometry operating parameters were: capillary voltage of 3 kV and sampling cone voltage of 10 V; desolvation gas flow of 1,000 L/h; and a temperature of 500°C. The source temperature was set at 150°C. The MRM mode was used to acquire the data, and the ion pairs were 496.3>184.1 for LPC (16:0); 524.4>184.1 for LPC (18:0); 522.4>184.1 for LPC (18:1); 762.5>184.1 for PC(16:0/18:0); and 760.5>184.1 for PC(16:0/18:1). The cone voltage was 10 V and the collision energy was 30 V. The data were analyzed with TargetLynx, including their integration, standard curve construction, etc.

### Detection of the Role of Angustoline in Regulating LKB1, AMPK, ELAVL1, and LPCAT2

Sixty samples (50 mg / sample) including 30 esophageal tumor tissue and 30 adjacent paracancerous tissue (normal control), were frozen in liquid N_2_ and homogenized rapidly, and then treated with protein lysis buffer (Solarbio) and centrifuged at 10,000 g (4°C, 10 min). Cell samples were prepared as follows, the KYSE450 cells were seeded into six-well plates for 24 h to achieve 25% confluence. LKB1 siRNA (10 μM), AMPK activator (10 μM), LPCAT2 siRNA (10 μM), AMPK siRNA (10 μM) and angustoline (1 mg/L) were added into the wells, respectively. siRNA fragment treatment complex was prepared as follows, (1) Transfectant reagent (4 μL/well, Santa Cruz Biotechnology) was diluted in 1 mL of fresh 1,640 medium. (2) The siRNA fragment (4 μg/well) was diluted in 1 mL of fresh medium. (3) The compounds in (1) and (2) were mixed together and the DNA-liposome complex (2 mL per well) was added into the cells. The samples were incubated at 37°C for 24 h, and then the DMEM-DNA-liposome mixture was added to the medium and cultured for another 24 h. The cell samples were collected and lysed with protein lysis buffer (Solarbio).

After 90°C heat treatment for 5 min, the protein samples were subjected to 12% SDS-polyacrylamide gel electrophoresis, and the samples were then transferred onto nitrocellulose membranes by Trans-Blot machines (Tanon, Shanghai, China). The membranes were blocked with 2% BSA buffer for 1.5 h at 25°C. LKB1, AMPK, ELAVL1, LPCAT2, and β-actin proteins were then probed with primary antibodies for 4 h at 25°C. β-actin was used as the internal reference to ensure equal loading. After three washes with TBST buffer (Solaribio, 6 min × 3), the membrane was incubated with secondary antibodies at 25°C for 2.5 h and then washed (7.5 min × 4). The protein bands in membranes were finally captured with ECL reagent (Tanon) and analyzed by Image J software (National Institutes of Health, Bethesda, MD, USA).

### Nude Mouse Xenograft Model

30 female BALB/c nude mice (18–20 g) were purchased from Beijing Vital River Laboratory Animal Technology Co., Ltd. (Beijing, China), and were fed in cages at 25°C with a relative humidity of 55%. The mice were acclimatized for at least 5 days before commencement. All procedures for animal experimentation were performed according to Chinese guidelines for animal care, confirming to internationally accepted principles in the care and use of experimental animals (NIH publications No. 8,023, in 1978). Animal experiments were approved by the Ethics Committee of Chinese Academy of Agriculture Sciences (Beijing, China), with the permission code of “CAS20190315 (Date: 15/03/2019).” The mice were randomly divided into six groups, control group (xenograft group), AMPK antibody treatment group, AMPK activator treatment group, angustoline treatment group, (AMPK antibody + angustoline) treatment group, (AMPK activator + angustoline) treatment group, *n* = 5. KYSE450 cells were cultured in a large scale, then 5 × 10^6^ cells in 200 μL matrigel medium (Corning) were subcutaneously injected into the right flank of each nude mouse in xenograft group. When the tumor volume reached 90–100 mm^3^, the mice in different groups were administered with AMPK antibody (1 mg/kg b.w.), AMPK activator (1 mg/kg b.w.), angustoline (10 mg/kg b.w.), AMPK antibody (1 mg/kg b.w.) + angustoline (10 mg/kg b.w.) and AMPK activator (1 mg/kg b.w.) + angustoline (10 mg/kg b.w.) by tail intravenous injection every 2 days. From the first day of administrations, all mice were sacrificed on the 25th day, the esophagus tissue in control group as well as tumors were separated out. All surgery was performed under sodium pentobarbital anesthesia, and all efforts were made to minimize suffering. Tumor diameters were recorded with a caliper every 4 days, and tumor volume was calculated using the following formula: tumor volume (mm^3^) = 0.5 × width (mm)^2^ × length (mm). Individual tumor suppression rate (%) = (the average tumor weight in the control group—the individual tumor weight in the lactoferrin treatment groups)/the average tumor weight in the control group × 100%, and the average tumor weight in the control group was calculated by each tumor weight in the control group. Relative tumor volume (RTV, %) = detected volume/volume before dosing × 100%. Relative tumor proliferation rate (%) = RTV of each tumor in the kinases or antibodies treatment groups/the average RTV in the control group × 100%, and the average RTV in the control group was calculated by RTV of each tumor in the control group. Mouse blood samples were collected, PC (16:0/18:1) and LPC (16:0) were detected by with LC-MS/MS.

### Data Analysis

All the data were presented as means ± standard deviations (SD). All data analyses were performed with the GraphPad Prism 7.0 software (GraphPad, San Diego, CA, USA). Statistical analyses were performed with Student's *t*-test and one-way analysis of variance (ANOVA). In the experiments based on ELISAs or western blotting, a *p*-value of < 0.05 was considered to indicate a statistically significant difference between the control and other groups.

## Results

### Angustoline Inhibited the Viability, Migration, and Invasion of KYSE450 Cells

**Figure 1:**
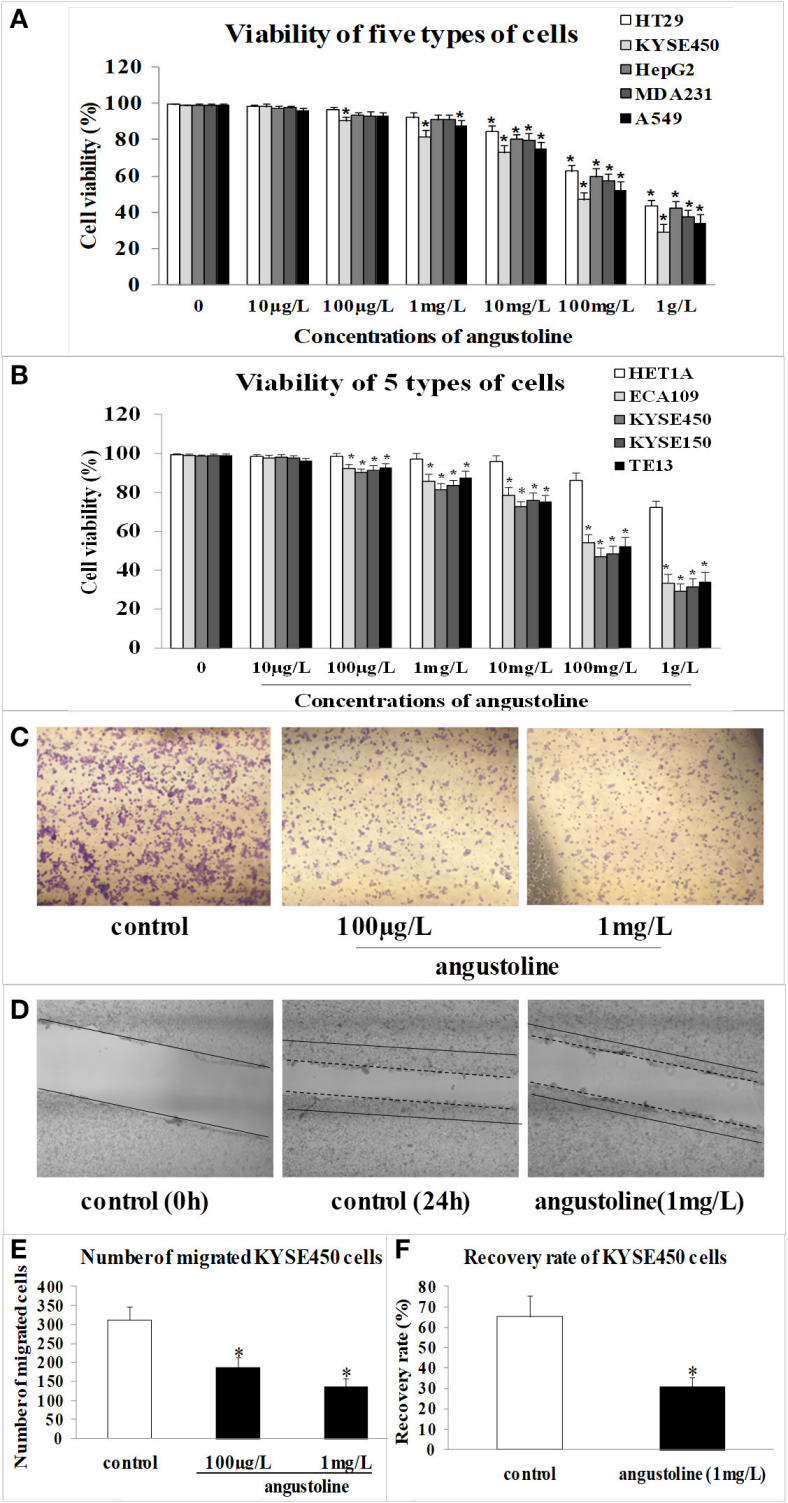
The effect of angustoline on the viability, migration and invasion of KYSE450 cells. **(A)** The viabilities of HT29 cells, KYSE450 cells, HepG2 cells, MDA231 cells, and A549 cells. **(B)** The viabilities of esophageal epithelial cells (HET1A), as well as several esophageal tumor cells, including, ECA109, KYSE450, KYSE150, and TE13. **(C)** The migration rate of KYSE450 cells in transwell assay. **(D)** The recovery rate of KYSE450 cells in scratch analysis assay. **(E)** Statistical analysis of the migrated cells. **(F)** Statistical analysis of the recovery rate. The data were represented as mean ± SD, *n* = 3. *p < 0.05, compared with the control. The photograghs were captured under 100 × magnification.

Through CCK8 detection, we found that angustoline inhibited viabilities of HT29 cells, KYSE450 cells, HepG2 cells, MDA231 cells, and A549 cells in different degrees, and the inhibition on KYSE450 cells was the highest one (Figure 1A). Further to compare the effect of angustoline on normal esophageal epithelial cells and esophageal tumor cells, we performed the viability detections in normal esophageal epithelial cell lines (HET1A), as well as in several esophageal tumor cell lines, including ECA109, KYSE450, KYSE150, and TE13. Results demonstrated that angustoline significantly inhibited the viabilities of the esophageal tumor cells, when compared with the normal esophageal epithelial cells (*p* < 0.05), and there seemed no obvious difference of cell viabilities among these esophageal tumor cells, indicating that esophageal tumor cells were sensitive to angustoline, when compared with esophageal epithelial cells (Figure 1B). Thus, esophageal tumor was selected as the sensitive one, KYSE450 cell was selected as the proper cell line and 1 mg/L was finally confirmed as the proper dose in the following experiments. In transwell and scratch analysis assays, angustoline with the dose of 1 mg/L was proved to inhibit migration and invasion of KYSE450 cells significantly (compared with the control, *p* < 0.05, Figures 1C–F). The above phenotypic experiment results indicated that angustoline could inhibit the growth and development of esophageal cancer.

### Two Special Lipid Metabolites Were Screened Out by Lipidomics Detections

**Figure 2:**
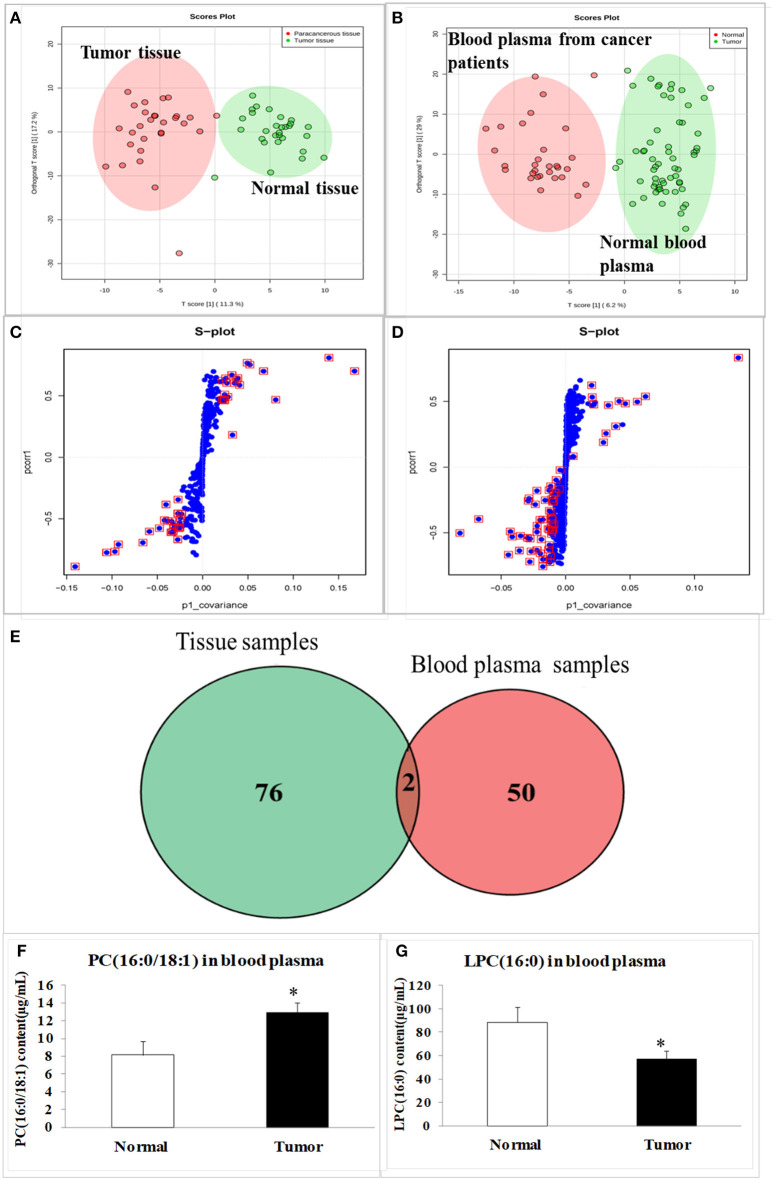
The scores plot and s-plot in clinical tissue samples and blood plasma samples, and the contents of special lipid metabolites in blood plasma samples. **(A)** The scores in tumor tissue and normal tissue from esophageal cancer patients; **(B)** The scores in blood plasma of esophageal patients and healthy volunteers; **(C)** The s-plot in tumor tissue and normal tissue; **(D)** The s-plot in blood plasma of esophageal patients and healthy volunteers; **(E)** The overlapped metabolites between tissue samples and blood plasma samples; **(F)** PC (16:0/18:1) content in blood plasma samples; **(G)** LPC (16:0) content in blood plasma samples. The data were represented as mean ± SD. In **(A–G)**, the total number (*n*) in tissue group or blood plasma group is 60, 30 normal samples, and 30 esophageal cancer patients samples, respectively. The data were represented as mean ± SD, *p < 0.05, compared with the control. *n* = 3.

The tissues of 30 esophageal cancer patients and their paracancerous tissues (considered to be as normal one), as well as the blood plasma of 30 healthy volunteers and 30 esophageal cancer patients were analyzed with a lipidomic approach using high-resolution quantitative time-of-flight (QTOF) mass spectrometry (MS). The data for all the samples were processed with an orthogonal partial least squares discriminant analysis (OPLS-DA) model. The scores plot indicated the clear separation of the lipidomic profiles of the esophageal cancer and normal subjects (Figures 2A,B), with good fits and predictive performances (R^2^Y = 0.52, Q^2^Y = 0.57 in tissues; R^2^Y = 0.71, Q^2^Y = 0.81 in blood plasma). We combined a variable importance in the projection (VIP) value of > 1 (Figures 2C,D, s-plot, red circles) and *p* < 0.05 to identify 78 different lipids in the tissues and 52 in the blood plasma (Figure 2E). The cross between tissue samples and blood plasma samples demonstrated that lysophosphatidylcholine (LPC; 16:0) and phosphatidylcholine (PC; 16:0/18:1) were the overlapped metabolites (Figure 2E). As quantifications of the two lipids by liquid chromatography (LC)-tandem MS (MS/MS) demonstrated, the average level of PC (16:0/18:1) in esophageal cancer patients was significantly higher than the one in healthy volunteers, while the level of LPC (16:0) in cancer patients was significantly lower than the healthy one (compared with the healthy control, *p* < 0.05, Figures 2F,G).

### The Role of Angustoline in Regulating LKB1/AMPK/ELAVL1/LPCAT2 Pathway Both in Human Tissues and KYSE450 Cells

**Figure 3:**
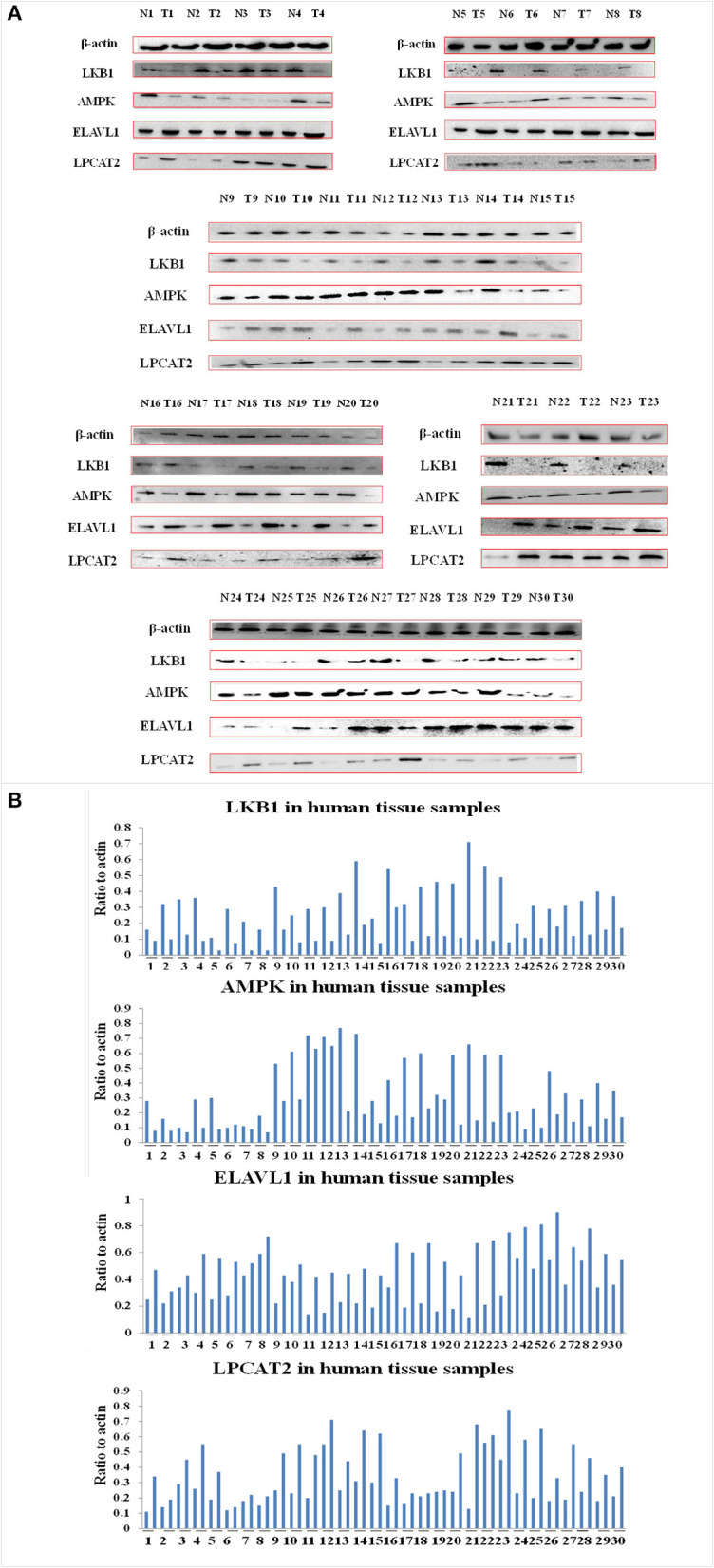
The levels of LKB1, AMPK, ELAVL1, LPCAT2, and β-actin proteins. **(A)** Expression of these proteins in 30 normal tissue samples and 30 esophageal cancer tissue samples. **(B)** Densitometric quantitations for normalized proteins relative to β-actin (%) in **(A)**.

**Figure 4:**
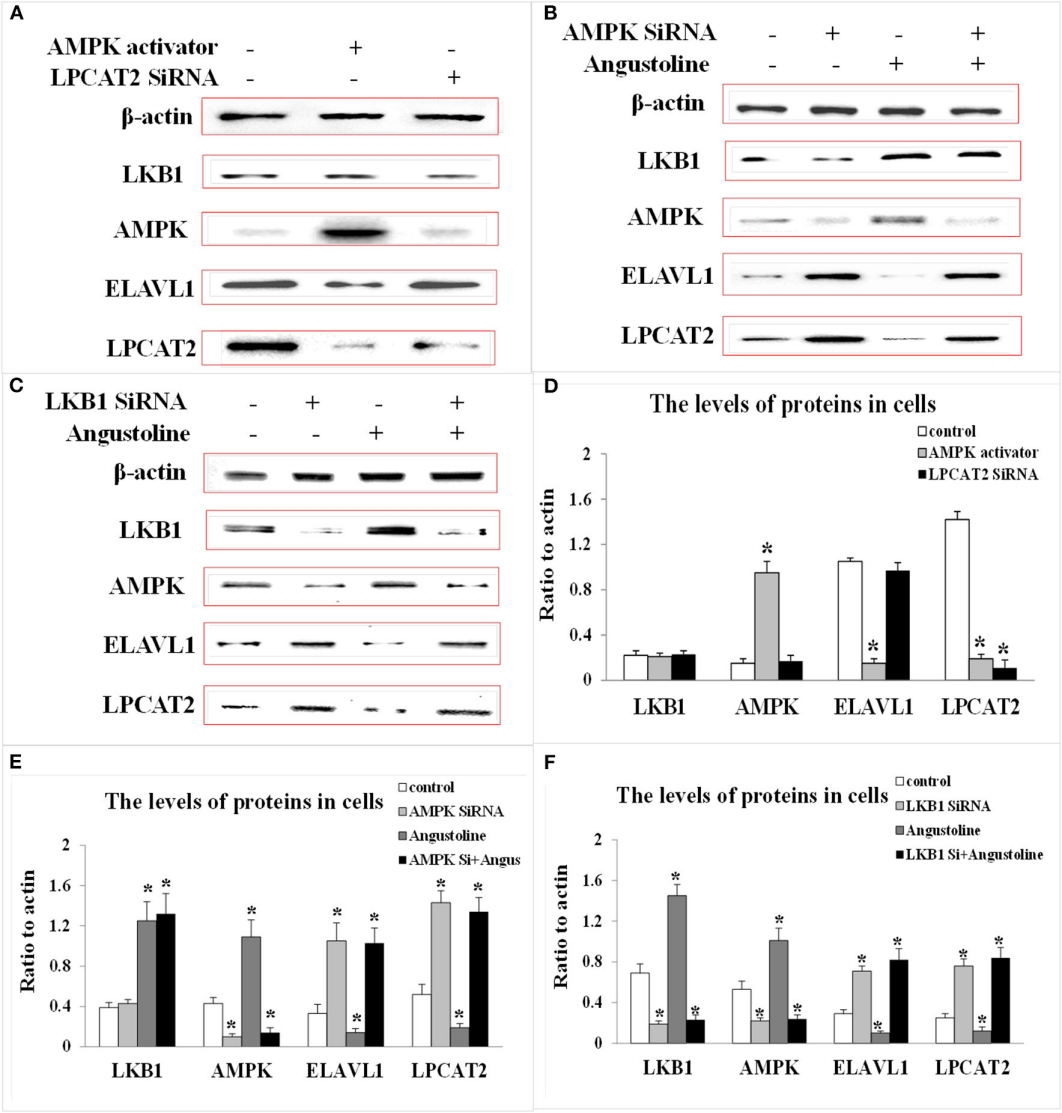
The levels of LKB1, AMPK, ELAVL1, LPCAT2, and β-actin proteins. **(A)** Expression of these proteins in KYSE-450 cells treated with AMPK activator or LPCAT2 siRNA fragment. **(B)** Expression of these proteins in KYSE-450 cells treated with AMPK siRNA or angustoline. **(C)** Expression of these proteins in KYSE-450 cells treated with LKB1 siRNA or angustoline. **(D)** Densitometric quantitations for normalized proteins relative to β-actin (%) in **(A)**. **(E)** Densitometric quantitations for normalized proteins relative to β-actin (%) in **(B)**. **(F)** Densitometric quantitations for normalized proteins relative to β-actin (%) in **(C)**. The data were represented as mean ± SD, *p < 0.05, compared with the control. *n* = 3.

To investigate the effect of angustoline on the molecular mechanism underlying lipids transformation in esophageal cancer, the protein levels of LKB1, AMPK, ELAVL1, and LPCAT2 were detected in 30 tumor tissues, as well as in the corresponding 30 paracancerous tissues. Results showed that the levels of AMPK and LKB1 in a large majority of the tumor samples were lower than the normal controls, the levels of ELAVL1 and LPCAT2 in tumor samples were higher than the normal control (*p* < 0.05, Figures 3A,B). Furthermore, to determine the role of AMPK in affecting ELAVL1 and LPCAT2, AMPK activator AICAR and LPCAT2-directed siRNA fragments) were utilized in KYSE450 cells, to investigate whether AMPK was the upstream regulator of ELAVL1 and LPCAT2. Results demonstrated that cytoplasmic ELAVL1 and LPCAT2 were downregulated in AMPK activator treatment group, and only the level of LPCAT2 was downregulated in LPCAT2 siRNA treatment group, when compared with the control (*p* < 0.05) (Figures 4A,D), proving that AMPK was the upstream negative regulator of ELAVL1 and LPCAT2, and LPCAT2 was the downstream sponsor of the other two factors. Additionally, LKB1 siRNA, AMPK siRNA, and angustoline were also utilized in KYSE450 cells, to prove the direct regulation of angustoline on AMPK and downstream factors through activating LKB1. The *in vitro* results showed that angustoline activated the expressions of LKB1 and AMPK and subsequently inhibited the levels of ELAVL1 and LPCAT2 (Figures 4B,C,E,F), which elucidated the anti-tumor mechanism of angustoline in esophageal cancer cell model.

### The Mechanism of Esophageal Tumor Growth Profiles Was Validated in Nude Mouse Xenograft Model

**Figure 5:**
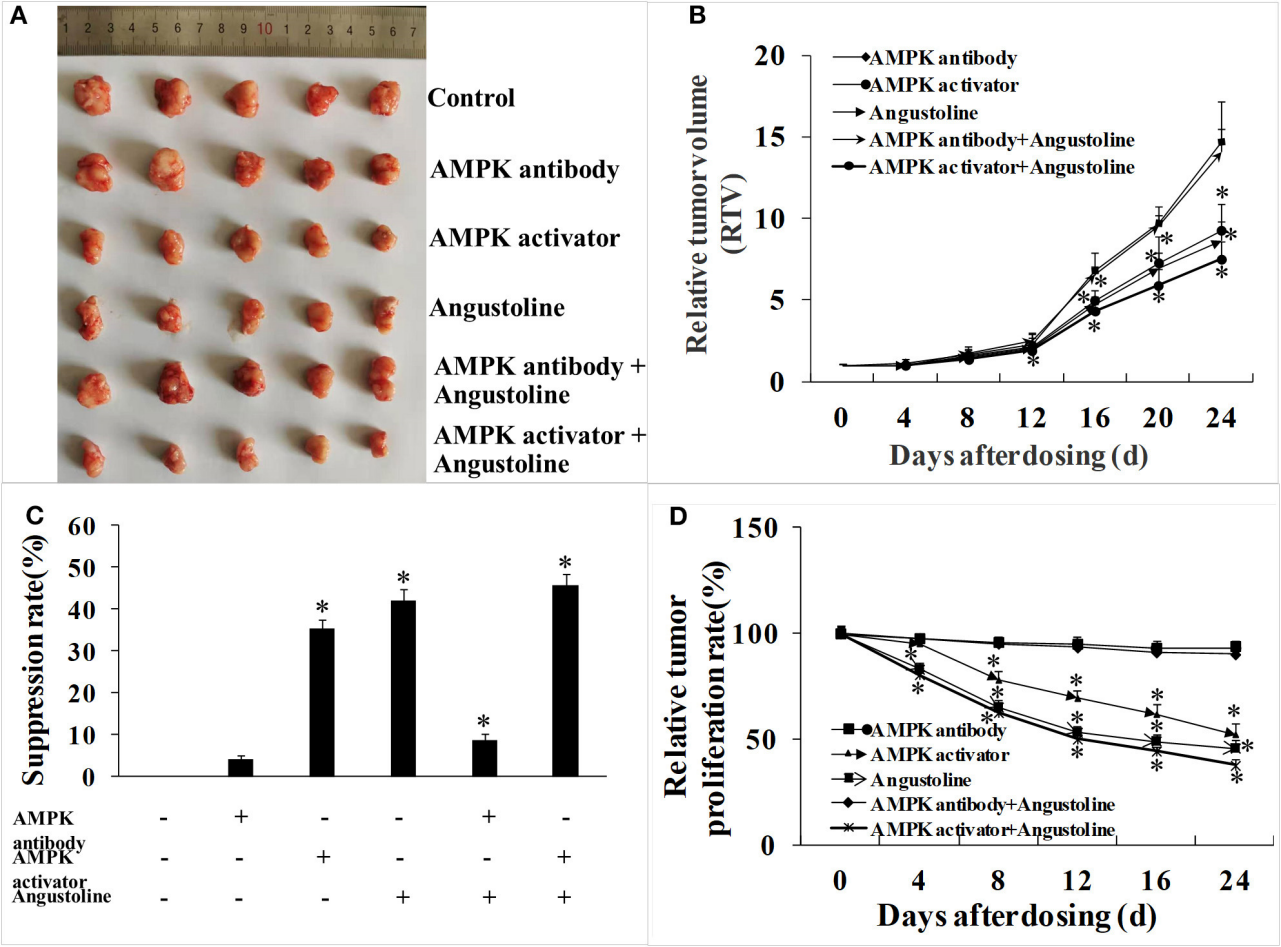
*In vivo* effects of AMPK activator/AMPK antibody/Angustoline on KYSE450 tumor-bearing nude mice. **(A)** Treatment of AMPK antibody/AMPK activator/Angustoline/(AMPK antibody + Angustoline)/(AMPK activator + Angustoline) on the size of KYSE450 tumors. **(B)** Relative tumor volume, which was calculated by each tumor volume. *p < 0.05, comparing with the control, *n* = 5. **(C)** Tumor suppression rate, which was calculated by each tumor weight. *p < 0.05, comparing with the control, *n* = 5. **(D)** Relative tumor proliferation rate, which was calculated by relative tumor volumes of different groups. *p < 0.05, comparing with the control, *n* = 5.

Data from the tumor-bearing nude mice model suggested that treatments of AMPK activator, angustoline, and (AMPK activator + angustoline) inhibited the growth and development of KYSE450 tumors implanted in nude mice. As shown in Figure 5, the relative tumor proliferation rate and relative tumor volume in the above three groups were reduced obviously, when compared with the control groups (*p* < 0.05) (Figures 5B,D). On the 25th day, KYSE450 tumor weights in all the treatment groups showed 2.41 ± 0.17 g, 2.30 ± 0.11 g, 1.55 ± 0.18 g, 1.39 ± 0.24 g, 2.19 ± 0.15 g and 1.32 ± 0.27 g, respectively (Figure 5C). Additionally, there seemed no obvious difference of tumor weights in control group, AMPK antibody group and (AMPK antibody + angustoline) group, indicating angustoline took anti-tumor effects mainly through activating the expression of AMPK.

### The Effect of Angustoline on the Levels of PC (16:0/18:1) and LPC (16:0)

**Figure 6:**
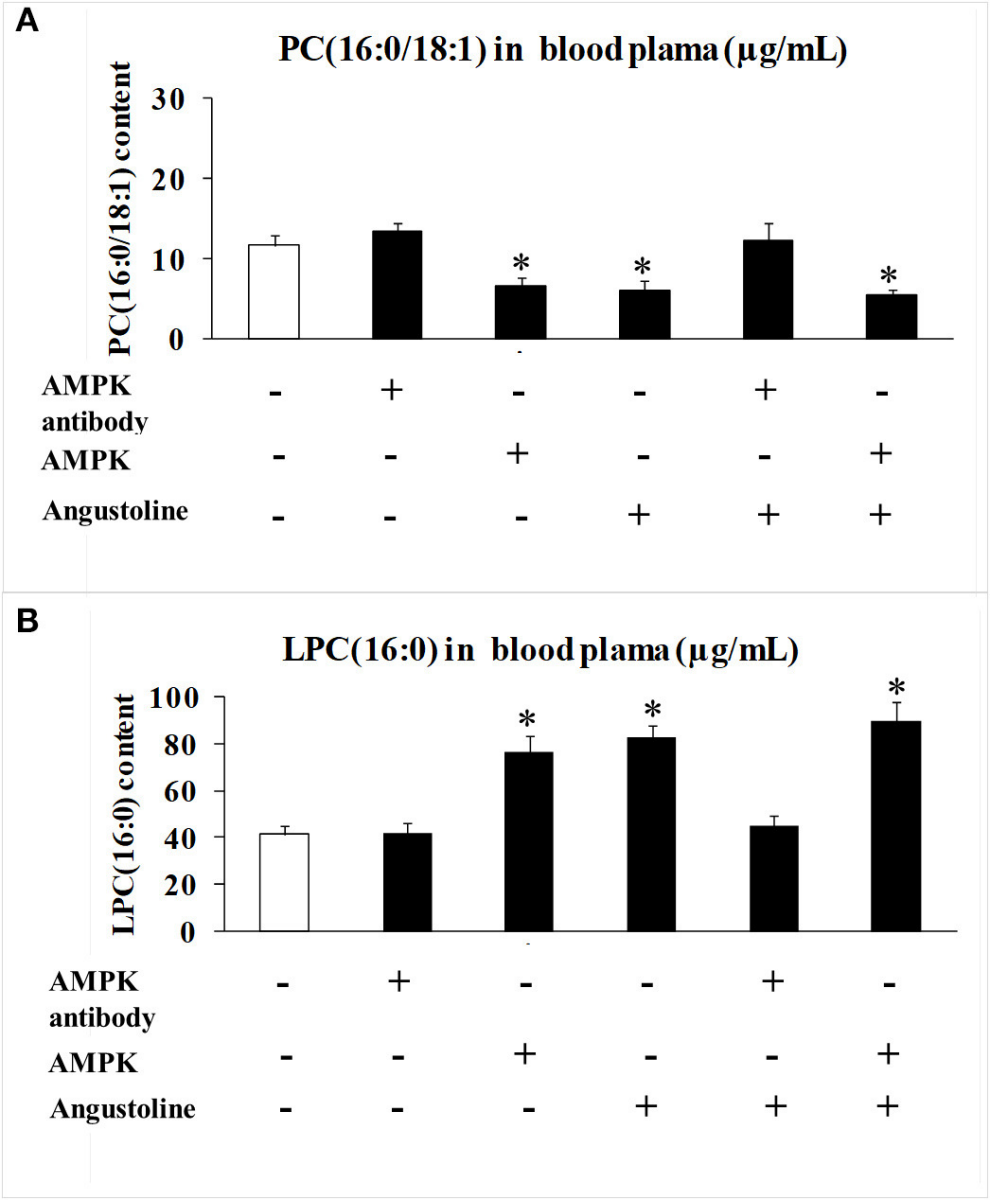
PC (16:0/18:1) and LPC (16:0) content detected by HPLC MS/MS analysis in mice blood plasma. **(A)** PC (16:0/18:1) content in mice blood plasma samples; **(B)** LPC (16:0) content in mice blood plasma samples. The data were represented as mean ± SD (*n* = 5). *p < 0.05, comparing with the control.

Further to observe the effect of angustoline on phospholipid remodeling, the levels of PC (16:0/18:1) and LPC (16:0) in mice blood plasma were detected. Results demonstrated that with the treatments of AMPK activator, angustoline or (AMPK activator + angustoline), PC (16:0/18:1) was downregulated and LPC (16:0) was upregulated when compared with the control (*p* < 0.05, Figure 6), validating that angustoline could suppress esophageal tumor through activating AMPK and inhibiting ELAVL1/LPCAT2, which consequently affecting phospholipids remodeling.

## Discussion

Cancer is currently considered to be a metabolic disease (2). Metabolomics (of which lipidomics is a branch), is a promising field of systems biology that investigates the sets of metabolites and lipids present in biological systems. Therefore, metabolomics and lipidomics have been used to study the biomarkers of many human diseases, including esophageal cancer (3–12). In this study, through primary screening of sensitive cancer cell lines, esophageal cancer was selected, and an untargeted lipidomic analysis based on an LC-MS/MS analysis of clinical samples (blood plasma samples from healthy volunteers and esophageal cancer patients, tumor tissues and paracancerous tissues from esophageal cancer patients), were performed to identify candidate lipid biomarkers in esophageal cancer. The special lipids PC (16:0/18:1) and LPC (16:0) were screened out as the candidate metabolites that differed significantly between esophageal cancer samples and normal subjects, and lipidomics was proved to be an excellent method for distinguishing esophageal cancer.

As an indole alkaloid, angustoline (C_20_H_17_N_3_O_2_, m.w. 331.37) also named vinmajine I, is isolated from the stems and leaves of *Nauclea officinalis*. Referring to its biological activities, there were several studies about it anti-inflammatory and antimalarial activity. Liu et al. (13) proved that angustoline showed a significant inhibitory activity on nitric oxide production induced by lipopolysaccharide in mouse macrophage RAW 264.7 cells. Sun et al. (14) found that indole alkoloids from *Nauclea officinalis*, including angustoline, took a weak antimalarial activity in plasmodium falciparum infected model. In colorectal cancer cells (LOVO), lung cancer cells (A549) and liver cancer cells (HepG2), angustoline showed very weak cell toxic effects (13). Though the research related with its anti-tumor effects was few, several analogs of angustoline were proved to demonstrate anti-tumor effects, like two diastereoisomeric 3,14-dihydroangustolines could inhibit the proliferation of bladder cancer cells (T-24) (15), as well as subditine, a new monoterpenoid indole alkaloid from bark of *nauclea subdita (Korth.) Steud*., could induce apoptosis of human prostate cancer cells (LNCaP and PC-3) (16). Considering the study on the effects of angustoline on esophageal tumor was rarely reported, the present manuscript investigated anti-tumor role of angustoline *in vitro* and *in vivo*, and explored the regulation of angustoline in LKB1/AMPK/ELAVL1/LPCAT2 pathway and phospholipid remodeling.

According to KEGG map of AMPK signaling pathway, serine/threonine-protein kinase 11 (also named LKB1) is one of the upstream factors of AMPK. LKB1 is a type of tumor suppressor gene and relatively highly expressed in esophageal tissue, which directly activates AMPK catalytic subunit PRKAA1, PRKAA2, and thereby regulates other downstream processes. The protein level of LKB1 decreased throughout prostate carcinogenesis, with a significant reduction already evident in high-grade prostate intraepithelial neoplasia lesions and a complete loss in adenocarcinomas (17). Studies demonstrated that ~30% of sporadic breast cancer samples expressed low levels of LKB1, yet overexpression of LKB1 protein was associated with a decrease in tumor micro vessel density (18). Similarly, researchers found that LKB1 inhibited proliferation of HeLa cell through activating AMPK, which subsequently inhibited the development of cervical carcinomas (19).

AMPK was known to play roles in the growth and metastasis of several types of cancers, including thyroid cancer, prostate cancer, ovarian cancer, lung cancer, etc., proving that AMPK could inhibit tumor cell growth and promote cell metastasis *in vivo* and *in vitro* (20–22). It was reported that the inactivation of AMPK prompted the growth and development of thyroid tumors, and the AMPK-activator (AICAR) could inhibit the basal and the TNF α-induced CXCL8 secretion, both in normal human thyroid cells and in thyroid cancer cell lines (20). Zhou et al. ever found that the inhibition of AMPK accelerated cell proliferation and promoted malignant behavior such as increased cell migration and anchorage-independent growth, and as a prototypical AMPK activator, AICAR caused the opposite changes in prostate cancer models (21). O'Brien et al. (22) also proved that salicylate could activate AMPK and synergize with metformin to reduce the survival of prostate and lung cancer cells *ex vivo* through inhibition of *de novo* lipogenesis. However, the investigation of AMPK in esophageal cancer models was rarely seen. Thus, we selected AMPK as a candidate regulator in the growth and metastasis of esophageal tumors in the present study. Human antigen R (HuR) is a member of the embryonic lethality-abnormal vision (ELAV) gene family and is also known as ELAVL1, which was widely involved in the regulation of gene transcription as it being a mRNA binding protein (23). Of the cis-acting elements of eukaryotic mRNA, the most characteristic is the ARE element (AU-rich element), ELAVL1 can increase the stability of its target mRNA by binding to the ARE elements located on the 3'-untranslated region (UTR) of a number of unstable mRNAs (23). Under normal physiological conditions, ELAVL1 is mainly localized to the nucleus, however, under conditions of stress, ELAVL1 binds to its target mRNA to form a complex and is shuttled to the cytoplasm, thus protecting its bound mRNA from degradation (24). Donahue ever found that in the absence of p53, ELAVL1 overexpression resulted in increased survivin mRNA stability and protein expression, which provided an additional explanation for the increased survivin expression observed in esophageal cancer cells that have lost p53 (25). A clinicopathological study performed by Zhang et al. (26) showed that cytoplasmic ELAVL1 expression was positively associated with lymph node metastasis, depth of tumor invasion, and advanced stage, whereas nuclear ELAVL1 expression was not correlated with any clinicopathological factors. Xu et al. (27) proved that ELAVL1 played a key role in the progression of esophageal carcinoma by targeting IL-18, which might be a potential therapeutic target for the treatment of ESCC. Referring to the roles of LPCATs in tumors, LPCAT1 were always applied and investigated in tumor models, results showed that the levels of LPCATs were sharply upregulated in colorectal cancer, prostate cancer, etc., which suggested LPCAT1 prompted the growth and metastasis of tumors (28, 29). However, the role of LPCAT2 in esophageal tumor model was rarely reported. Therefore, LKB1, AMPK, ELAVL1, and LPCAT2 were chosen as the possible candidate factor in esophageal tumor models in our study, their roles in the growth and development of esophageal tumor, as well as the effect of angustoline on their expression levels, were investigated.

With the transfection of LKB1 siRNA, LPCAT2 siRNA, or AMPK activator, we proved that LKB1 was the upstream regulator of AMPK and AMPK was the upstream factor of ELAVL1 and LPCAT2, with the treatment of LKB1 siRNA, AMPK siRNA, and angustoline, we further validated that angustoine inhibited esophageal tumor through activating LKB1 and AMPK, then suppressing ELAVL1, and LPCAT2.

Phospholipids and LPCATs are reported to be key factors in cell growth, tumor progression, and cancer aggressiveness, especially LPCAT1 (28, 29), but only a few studies have investigated the roles of LPCAT2 in regulating phospholipids only in colorectal cancer (CRC) patients (30, 31). No direct evidence or a fully comprehensive mechanism has yet been reported. Here, for the first time, we had used siRNA fragments to provide a mechanism that linked ELAVL1 with phospholipid remodeling, proving ELAVL1 was involved in the overexpression of LPCAT2, which further catalyzed the conversion of LPC (16:0) to PC (16:0/18:1) in esophageal cancer cells.

Further to verify the roles of LKB1, AMPK, ELAVL1, and LPCAT2 in the development of esophageal cancer, as well as the anti-tumor effect of angustoline in esophageal tumor model, we also utilized AMPK antibody and AMPK activator to treat the KYSE450 tumor bearing nude mice, and found that the tumor weights, relative tumor volume and tumor proliferation rate were significantly inhibited with the treatments of AMPK activator or angustoline, when compared with the non-treatment control. The results further confirmed that angustoline suppressed esophageal tumors through regulating LKB1/AMPK/ELAVL1/LPCAT2 and consequently inhibiting the excessive conversion from LPC (16:0) to PC (16:0/18:1). Therefore, the development of angustoline or AMPK inhibitor as novel anti-tumor drugs or adjunctive therapy drugs, provided novel treatment plans in clinical esophageal cancer field. Additionally, two factors, LPC (16:0) and PC (16:0/18:1) were determined to participate in the growth and development of esophageal tumors, which might be developed as clinical prognostic indicators, and predict the patient's response to conventional neoadjuvant therapies or to the more recently described immunotherapies for advanced-stage disease.

## Data Availability Statement

The datasets generated for this study can be found in FigShare (https://figshare.com/s/18ab289df8c8da53b26c).

## Ethics Statement

The studies involving human participants were reviewed and approved by Ethics Committee approval of the First Affiliated Hospital of Chongqing Medical University. The patients/participants provided their written informed consent to participate in this study. The animal study was reviewed and approved by Ethics Committee of Chinese Academy of Agriculture Sciences (Beijing, China). Written informed consent was obtained from the individual(s) for the publication of any potentially identifiable images or data included in this article.

## Author Contributions

HL, CZ, and MZ performed the experiments, analyzed the data, and prepared the manuscript draft. CZ and MZ provided the human specimens. HL and QY set up the experiments and repeated the key experiments. HY and LF conceived the work, analyzed the data. HL and NZ prepared the manuscript. All authors contributed to the article and approved the submitted version.

## Conflict of Interest

The authors declare that the research was conducted in the absence of any commercial or financial relationships that could be construed as a potential conflict of interest.
