# Supplementary material for: Angustoline Inhibited Esophageal Tumors Through Regulating LKB1/AMPK/ELAVL1/LPACT2 Pathway and Phospholipid Remodeling
Source: Front Oncol. 2020 Jul 7;10:1094. doi: 10.3389/fonc.2020.01094 (PMC7358378; doi:10.3389/fonc.2020.01094)
Supplement: Supplementary file 1 [file Data_Sheet_1.DOCX]

**Procedure of ultrahigh-performance LC-(UPLC)-QTOF-MS in lipidomics detection**

The UPLC-QTOF-MS analysis was performed with the Acquity UPLC® *I*-Class System (Waters), coupled to an electrospray ionization (ESI)-QTOF mass spectrometer (Xevo® G2-S Q-Tof, Waters). A reversed-phase LC column (Acquity UPLC CSH C18 Column, 130 Å, 1.7 µm, 2.1 mm × 100 mm) was used for the separation with two solvents: ‘A’ containing acetonitrile and water (3:2) with 10 mM ammonium formate and 0.1% formic acid; and ‘B’ containing isopropanol and acetonitrile (9:1) with 10 mM ammonium formate and 0.1% formic acid. The UPLC autosampler temperature was set at 10 °C and the injection volume for each sample was 2 μL. The column temperature was maintained at 55 °C.

Mass spectrometry was performed in either the positive (ESI+) or negative (ESI-) electrospray ionization mode. The data-independent MS^E^ scan method of the Waters QTOF mass spectrometer was used, with which both the high-accuracy primary MS and secondary MS/MS data for the precursor ions can be automatically acquired. The MS^E^ data were acquired in the continuum mode in a mass range from 50 Da to 1500 Da for TOF-MS scanning. A capillary voltage of 3 kV and a sampling cone voltage of 30 V were used in both ionization modes. The desolvation gas flow was set to 500 L/h, and the temperature was set to 400 °C. The source temperature was set to 120 °C. The MS^E^ scan was performed in two scan functions using ramp collision energy. For the low-energy mode, a scan range of 100-1500 Da, a scan time of 0.2 s, and a collision energy of 6 V were used. For the high-energy mode, a scan range of 50-1500 Da, a scan time of 0.2 s, and a collision energy ramp of 15-45 V were used. The accurate mass was maintained by introducing a lock-spray interface of leucine–enkephalin (556.2771 [M+H]^+^ or 554.2615 [M-H]^−^) at a concentration of 1 pg/µL at a rate of 5 µL/min. Pooled quality control samples (generated by combining equal aliquots of all the samples included in the experiment) were run at the beginning of the sample queue to condition the column and after every 10^th^ injection thereafter to correct the drift in the retention times and the variation in the ion intensity over time. The sample queue was randomized to remove any bias. Batch acquisition was repeated to check the experimental reproducibility.
